# Sex differences in the temporal dynamics of autistic children’s natural conversations

**DOI:** 10.1186/s13229-023-00545-6

**Published:** 2023-04-06

**Authors:** Sunghye Cho, Meredith Cola, Azia Knox, Maggie Rose Pelella, Alison Russell, Aili Hauptmann, Maxine Covello, Christopher Cieri, Mark Liberman, Robert T. Schultz, Julia Parish-Morris

**Affiliations:** 1 Linguistic Data Consortium, University of Pennsylvania, 3600 Market Street, Philadelphia, PA 19104 USA; 2 Center for Autism Research, Children’s Hospital of Philadelphia, Philadelphia, PA 19104 USA; 3 Department of Psychology, La Salle University, Philadelphia, PA 19141 USA; 4 Department of Psychiatry, Perelman School of Medicine at the University of Pennsylvania, Philadelphia, PA 19104 USA; 5 Department of Pediatrics, Perelman School of Medicine at the University of Pennsylvania, Philadelphia, PA 19104 USA

**Keywords:** Autism, Natural language, Conversation, Sex differences, Speech rate, Speech duration, Interruption, Prosody, Temporal dynamics

## Abstract

**Background:**

Autistic girls are underdiagnosed compared to autistic boys, even when they experience similar clinical impact. Research suggests that girls present with distinct symptom profiles across a variety of domains, such as language, which may contribute to their underdiagnosis. In this study, we examine sex differences in the temporal dynamics of natural conversations between naïve adult confederates and school-aged children with or without autism, with the goal of improving our understanding of conversational behavior in autistic girls and ultimately improving identification.

**Methods:**

Forty-five school-aged children with autism (29 boys and 16 girls) and 47 non-autistic/neurotypical (NT) children (23 boys and 24 girls) engaged in a 5-min “get-to-know-you” conversation with a young adult confederate that was unaware of children’s diagnostic status. Groups were matched on IQ estimates. Recordings were time-aligned and orthographically transcribed by trained annotators. Several speech and pause measures were calculated. Groups were compared using analysis of covariance models, controlling for age.

**Results:**

Autistic girls used significantly more words than autistic boys, and produced longer speech segments than all other groups. Autistic boys spoke more slowly than NT children, whereas autistic girls did not differ from NT children in total word counts or speaking rate. Autistic boys interrupted confederates’ speech less often and produced longer between-turn pauses (i.e., responded more slowly when it was their turn) compared to other children. Within-turn pause duration did not differ by group.

**Limitations:**

Our sample included verbally fluent children and adolescents aged 6–15 years, so our study results may not replicate in samples of younger children, adults, and individuals who are not verbally fluent. The results of this relatively small study, while compelling, should be interpreted with caution and replicated in a larger sample.

**Conclusion:**

This study investigated the temporal dynamics of everyday conversations and demonstrated that autistic girls and boys have distinct natural language profiles. Specifying differences in verbal communication lays the groundwork for the development of sensitive screening and diagnostic tools to more accurately identify autistic girls, and could inform future personalized interventions that improve short- and long-term social communication outcomes for all autistic children.

**Supplementary Information:**

The online version contains supplementary material available at 10.1186/s13229-023-00545-6.

## Introduction

Autism is a heterogeneous neurodevelopmental condition characterized by repetitive patterns of behavior, restricted interests [1], and social communication challenges that result in functional impairment [2]. Autism affects 1 in 44 youth in the USA [3] and is diagnosed more frequently in boys than in girls. The most replicated sex ratio is 4:1 [4], yet the ratio ranges from 2:1 to 7:1 depending on which diagnostic procedures are used [5–8]. Research shows that autistic girls and women are more likely than autistic boys and men to be missed or diagnosed late [4], even when they have comparable clinical symptoms [9]. One possible reason for this diagnostic gap is that verbally fluent autistic girls make significantly better first impressions than autistic boys during brief naturalistic “get-to-know-you” conversations [10], which could reduce referral rates and complicate diagnosis. “Social camouflage” has been implicated in the literature as a potential reason for missed diagnoses in girls [11], but the objective behavioral mechanisms that underlie sex differences in first impressions are just beginning to be understood. In this study, we examine sex differences in verbal communication during naturalistic conversations which—when combined with traditionally male-referenced diagnostic criteria—could contribute to systematically late or missed diagnoses in autistic girls [3, 4, 9, 12, 13].

### Sex differences in autism

The primary features of autism are consistent across sexes (i.e., social communication challenges and the presence of restricted and repetitive patterns of behaviors/interests), but the specifics of how these symptoms manifest in the real world have been shown to differ. In the past, studies relying on predominantly male samples were often expected to generalize to all autistic people [14], resulting in the misconception that autism can always be identified using male-typical patterns. For example, Klin and colleagues [15] studied restricted interests in 91 boys and 5 girls with autism, drawing the conclusion that *autism* is associated with extreme *common male interests* such as constructing Legos and making models of cars. Most of the existing literature in autism includes sex-imbalanced studies, leading to concerns that the historical conceptualization of autism is confounded with “maleness” in a way that impairs our ability to identify and support autistic girls and women [16].

Compared to boys, girls with autism present distinct symptom profiles across a variety of domains. For instance, autistic girls show either fewer or harder-to-detect repetitive behaviors and interests—which are also more similar in topic to the interests of same-sex neurotypical (NT) peers—relative to autistic boys [5, 17, 18]. Autistic girls without co-occurring intellectual disability demonstrate greater social motivation and better friendship quality than age- and IQ-matched autistic boys [19], and eye tracking research has also shown that autistic girls pay more attention to faces and social images than autistic boys, and both sexes prefer looking at toys that fall along traditional gender lines [20–22].

Girls and women with autism may also employ effortful camouflaging or compensation strategies to mask their autism symptoms, and report doing this to a greater extent than boys and men [23]. In addition, autistic girls and women have been shown to exhibit nonverbal behaviors that are more similar to their NT peers during social interaction, such as mimicking others’ facial expressions or gestures and making more frequent eye contact [11, 12]. During recess, girls with autism tend to hover near groups of other girls, whereas autistic boys tend to be more socially isolated [16]. In clinical settings, autistic girls have been found to use more social and friend-related words during diagnostic assessments compared to autistic boys, despite being matched on age, IQ, and autism symptom severity [24]. However, long-term effortful social camouflaging to “fit in” or appear less autistic may result in poor mental health for individuals with autism [11] and can contribute to autistic burnout [25].

Lack of understanding about autistic girls and women has serious downstream consequences. Systematic underdiagnosis and misdiagnosis results in missed opportunities to provide tailored, personalized interventions, leaves girls and women with reduced access to social supports, and increases the likelihood of experiencing social rejection, sexual abuse, and poor mental health outcomes [23, 26, 27]. A specific focus on characterizing verbal communication patterns in autistic girls and boys during naturalistic conversations could improve diagnostic accuracy and ultimately inform the development of supports that are tailored to the needs of autistic girls and women. In this study, we explored the language profiles of autistic and NT girls and boys during naturalistic interactions with novel interlocutors, designed to simulate a “get-to-know-you” conversation.

### Language in autism

Language impairment is not a “core” feature of autism, but differences in language *use*—especially during everyday interactions—contribute to the social communication challenges that characterize autism. For example, a well-known diagnostic self-report questionnaire measuring autistic traits, the Autism-Spectrum Quotient Test (AQ, [28]), includes questions centered on language, such as “I frequently find that I don’t know how to keep a conversation going,” “I find it easy to read between the lines when someone is talking to me,” and “When I talk on the phone, I’m not sure when it’s my turn to speak.” Atypical language patterns have been described extensively in the autism literature, including descriptions of “awkward” prosody [29], unusual disfluency patterns [30], nonstandard pronoun use [31–34], impaired pragmatic skills [2], increased concrete and literal word use [35, 36], and decreased frequency of cognitive process words [37–39].

For verbally fluent autistic individuals, conversational language forms a critical pathway to friendships, romantic relationships, jobs, and overall quality of life [40]. In contrast to prior research focused on measuring language using standardized assessments or structured elicitations [41–43], more recent studies examined language during natural conversations. These naturalistic language samples—by virtue of more closely approximating the demands of real-world social situations—have enhanced ecological validity that could shed new light on whether and how the social phenotypes of autistic boys and girls differ in real-world contexts. Natural conversational samples also facilitate examination of intricate social dynamics, such as “who speaks when and for how long,” known as *durational measures*.

Studies that target acoustic and durational measures during natural conversations in autism have yielded mixed results, suggesting the need for further research to understand these key variables. For example, autistic children with more severe symptoms have been shown to produce decreased speech duration in both structured assessment settings [44] and unstructured conversational environments [45]. However, this finding was not replicated in young adults with autism [46] nor in infants with a high familial likelihood of autism [47], where high familial likelihood infants did not produce shorter vocalizations compared to the low familial likelihood group. Also, some prior studies observed that autistic children spoke more slowly than NT peers [48, 49], yet others failed to replicate this finding [50]. Only few studies have investigated the temporal dynamics of responsiveness during natural conversations, but autistic children’s longer latency to respond [51] has been frequently and consistently observed. Previous research has also shown that autistic children may produce either “sing-songy” or monotonous pitch contours [52, 53], which are opposite descriptions of prosody. There appears to be a consistent trend toward wider pitch variability in children with autism [49, 50, 54] (also see a meta-analysis in [29]), but some studies do not report this group difference [47, 55, 56].

Previous research has examined characteristics of autistic children’s language behavior during natural conversations, but the results are far from conclusive. Evidence for how autistic girls and boys may or may not differ from each other during natural conversations is particularly scarce, because most previous studies did not include sufficient numbers of girls with autism to assess sex differences. Two recent studies investigated sex differences in autism during natural conversations and found that autistic girls produced more words and speech than autistic boys [34] as well as fewer disfluencies [30]. This stands in sharp contrast to studies of non-autistic people, which did not reveal sex differences in either total number of words or total speech duration, with mixed results reported for filler counts [57, 58]. Importantly, studies of natural conversations in autistic children remain the exception rather than the rule, and rarely examine the distinctive effects of group and sex in durational and acoustic measures, leaving a significant gap in the literature.

To address this gap, we investigated sex differences in the verbal behavior of autistic children during brief, naturalistic “get-to-know-you” conversations. Specifically, we focused on temporal organization, such as speech/pause duration and overall amount of talking. This approach has the advantage of being topic-independent; that is, *how* children talk is far more important than *what* they talk about. Specifically, we hypothesized that autistic girls would speak more with a novel interlocutor than autistic boys, given previous research suggesting greater social motivation in autistic girls [19] and studies suggesting that differences in social motivation (or social focus) may be detectable in natural language samples [34]. We further hypothesized that conversations between autistic girls and interlocutors would include longer speech segments and more frequent instances of overlapping speech, in part due to research showing that successful conversations exhibit some degree of overlapping speech between conversational partners [59]. Lastly, we expected that autistic boys would pause longer before responding to interlocutors (i.e., demonstrate longer latency to respond), in light of prior studies on this topic with results that may have been driven by predominantly male research samples.

## Methods

### Participants

Forty-five school-aged children with autism (29 boys and 16 girls) and forty-seven NT children (23 boys and 24 girls) were drawn from a pool of individuals seen at a large academic medical research center (Children’s Hospital of Philadelphia Center for Autism Research). All participants completed a 5-min “get-to-know-you” conversation with a novel young adult confederate (*n* = 22, 19 females) during the research study visit. Participant sex was characterized using parent-reported sex assigned at birth. Children participated in a larger series of studies that included autism diagnostic assessments, IQ testing, and behavioral tasks.

Participants’ social and repetitive behavior symptoms were characterized using ADOS-2 Calibrated Severity Scores [60], the Social Communication Questionnaire (SCQ; [61]), and the Social Responsiveness Scale-2 (SRS-2; [62]). Autism diagnoses were made by expert PhD-level clinicians using the clinical best estimate (CBE) approach [63] informed by a research-reliable administration of the ADOS-2 [63]. The CBE method prioritizes DSM-5 criteria informed by family/medical history and an evaluation by an autism specialist. The Center for Autism Research does not rely solely on ADOS-2 or SCQ cutoff scores when diagnosing autism, nor do subthreshold scores lead to automatic exclusion. This is because many disorders can result in elevated scores on these metrics (e.g., ADHD [64]), and the behavior snapshot afforded by the ADOS-2 may not fully capture the scope or impact of an individual’s symptoms. Five autistic children (3 girls and 2 boys) scored in the low range for autism-related symptoms on the ADOS-2 calibrated total score (< 4; three children scored 2, and two children scored 3). However, those children were given an autism diagnosis since they met the DSM-5 criteria for clinician best estimate and the SCQ lifetime cutoff value (> 15, mean SCQ of the five children = 21.4, range = 16–31). For NT participants, autism was ruled out using an ADOS-2 administration, SCQ scores, and clinician judgment according to DSM-5 criteria. NT participants did not have any current DSM-5 diagnoses nor any clinically significant psychiatric or neurodevelopmental symptoms per parent report or clinical observation.

Participants were categorized into one of four groups by diagnostic status and sex: autistic girls, autistic boys, NT girls, and NT boys. To match groups, participants with complete data (age, sex, race, ADOS-2 (Module 3: *n* = 85, Module 4: *n* = 7), IQ estimates, and usable language samples) were drawn from the larger pool. Participants from the larger pool were excluded from the present analyses if they had a full-scale IQ or verbal IQ ≤ 70. The four groups were matched on their full-scale IQ estimates, verbal and nonverbal IQ estimates, as measured by the Differential Abilities Scales-II (DAS-II; [65]), the Wechsler Abbreviated Scale of Intelligence-2nd Edition (WASI-II; [66]), the Stanford-Binet Intelligence Scales-5th Edition (SB5; [67]), or the Wechsler Intelligence Scale for Children (WISC-V; [68]). Raw scores were standardized and transformed into full-scale, verbal and nonverbal IQ scores, respectively, by an expert neuropsychologist. Autistic boys were significantly older than NT boys (*p* = 0.026), NT girls (*p* = 0.029), and autistic girls (*p* = 0.043). Age was included as a covariate in all subsequent analyses.

Participants’ adaptive behavior was measured using the Vineland Adaptive Behavior Scales, 2nd edition [69]. Autistic boys and girls did not differ on the ADOS-2 total (*p* = 0.72), the ADOS social affect scores (*p* = 0.11), or ADOS-2 restricted and repetitive behavior (*p* = 0.1) scores. They were also matched on Vineland socialization (*p* = 0.36), communication (*p* = 0.31), daily living (*p* = 0.1), and composite scores (*p* = 0.16). The difference in SRS-2 scores between autistic boys and girls was marginally significant (*p* = 0.051), and it trended toward more autism symptoms in girls. NT girls and boys did not significantly differ in any of these clinical measures.

All participants were native speakers of English and verbally fluent according to the definition in the ADOS-2 manual. The total duration of the “get-to-know-you” conversations did not differ by group (*p* = 0.64). Participants’ demographic and clinical characteristics are summarized in Table 1.

**Table 1:** Demographic and clinical characteristics of participants

	Autistic boys (*N* = 29)	Autistic girls (*N* = 16)	*p*-value (autistic girls vs. autistic boys)	NT boys (*N* = 23)	NT girls (*N* = 24)	*p*-value (all group comparisons)
Age (years)	12.3 (3.1)	10.0 (1.5)	0.043	10.0 (2.8)	10.1 (2.9)	0.006
Race			0.213			0.122
*N*-missing	0	1		0	0	
African American or Black	2 (6.9%)	0 (0.0%)		7 (30.4%)	3 (12.5%)	
Asian or Pacific Islander	0 (0.0%)	1 (6.7%)		1 (4.3%)	1 (4.2%)	
Caucasian or White	26 (89.7%)	12 (80.0%)		12 (52.2%)	17 (70.8%)	
More than one race	1 (3.4%)	2 (13.3%)		3 (13.0%)	3 (12.5%)	
Full-scale IQ (DAS-II GCA)	103.9 (13.4)	109.2 (8.7)	0.155	108.4 (12.2)	108.7 (12.6)	0.363
Verbal IQ	104.2 (14.3)	106.9 (9.6)	0.502	106.8 (13.2)	110.1 (15.0)	0.471
Nonverbal IQ	102.8 (11.8)	109.1 (12.8)	0.105	107.9 (11.9)	104.4 (12.8)	0.286
Maternal education			0.176			0.136
N-Missing	6	3		1	0	
Bachelor’s or less	13 (56.5%)	4 (30.8%)		15 (68.2%)	12 (50.0%)	
Graduate degree	10 (43.5%)	8 (61.5%)		5 (22.7%)	12 (50.0%)	
High school or less	0 (0.0%)	1 (7.7%)		2 (9.1%)	0 (0.0%)	
ADOS-2 Total	6.5 (1.8)	6.2 (2.4)	0.715	1.3 (0.4)	1.1 (0.3)	< 0.001
ADOS-2 Social affect	6.9 (1.6)	5.9 (2.1)	0.105	1.7 (0.9)	1.5 (0.8)	< 0.001
ADOS-2 RRB	6.4 (2.3)	7.5 (1.6)	0.097	1.7 (1.9)	1.3 (1.1)	< 0.001
SCQ lifetime	18.5 (7.5)	18.2 (7.0)	0.897	3.0 (3.2)	1.8 (2.1)	< 0.001
Social Responsiveness Scale (total)	67.1 (9.4)	73.9 (11.0)	0.051	46.4 (7.3)	45.5 (5.5)	< 0.001
VABS Communication	83.9 (14.4)	88.4 (11.1)	0.306	108.0 (12.4)	110.1 (10.1)	< 0.001
VABS Socialization	75.9 (12.2)	79.6 (13.1)	0.362	105.3 (9.8)	106.4 (8.5)	< 0.001
Total conversation duration (sec)	323.6 (17.6)	323.8 (17.7)	0.975	326.1 (36.2)	333.3 (38.7)	0.642

Results of *t*-tests comparing autistic boys and girls are presented in the first *p*-value column. Results of the ANOVA models for the comparison of all four groups are shown in the second *p*-value column (autistic girls vs. autistic boys vs. NT girls vs. NT boys)

NT: neurotypical; ADOS-2: Autism Diagnostic Observation Schedule, 2nd Edition; RRB: Restricted and repetitive behaviors; SCQ: Social Communication Questionnaire; VABS: Vineland Adaptive Behavior Scales

### Procedure

Children had a short, unstructured conversation with a young adult research assistant (hereafter, confederate or interlocutor) in a quiet room at the Center for Autism Research, Children’s Hospital of Philadelphia. Confederates were undergraduate students or research assistants who were unaware of participants’ diagnostic status and were assigned to each participant based on scheduling availability. At the start of the conversation, a second research assistant in charge of the visit introduced the task using a variation of the phrase, “You two just chat and get to know each other. I’m going to finish getting a few things set up.” Confederates were instructed to speak for no more than 50% of the conversation, but given no other directions. Conversational prompts were not provided to either speaker. Conversations between children and confederates were audio/video-recorded using two small HD video cameras and four directional microphones placed, unobtrusively, on a table between the participant and the confederate so that the participant and confederate were simultaneously recorded as they sat facing each other during the conversation. Excerpts of sample conversations are provided in Additional file 1.

### Annotation

Audio signals were extracted from recordings, and saved in lossless.flac format, and later converted to.wav format with 16 kHz of sampling rate for audio processing. A team of trained annotators produced time-aligned, verbatim transcripts of audio recordings using an in-house annotation program developed at the Linguistic Data Consortium of the University of Pennsylvania. Each recording was processed by two junior annotators and one senior annotator, all of whom were undergraduate students and native English speakers. Annotators were instructed to segment speech (i.e., place time stamps) at the beginning and end of long pauses (150 ms) and at the moment of speaker changes, and to transcribe each speech segment one at a time. To become junior annotators for this project, each team member received at least 10 h of training in Quick Transcription [70] modified for use with clinical interviews of autistic participants. In addition, annotators had to achieve reliability (defined as > 90% in common with a gold standard transcript) on segmenting (marking start and stop times of speech segments) and transcribing (writing down words and sounds produced, using the modified Quick Transcription specification) before beginning independent annotation. One junior annotator segmented utterances into speech segments, and the second junior annotator transcribed words produced by each speaker. A senior annotator with at least 6 months of annotation experience then thoroughly reviewed and corrected each file. Final language data were exported as tab-delimited files.

### Measurements

#### Conversational turns

In this study, we defined “turns” based on speaker change. All utterances produced by one speaker, until a speaker change, were considered part of one turn. Utterances within turns were defined as “speech segments.” Using transcript files, we placed speech segments into of three categories: participant, confederate, or overlapping. Since data were drawn from natural conversations, we identified overlapping speech, where the duration from the end of the previous speech segment to the start of the next speech segment was negative (i.e., overlapping), and included the sum of the overlapping speech duration in the analysis. We further classified overlapping speech into two categories depending on who was interrupting the previous speaker. The sums of participants’ interrupting duration and confederates’ interrupting duration (i.e., when participants were interrupted) were calculated separately.

#### Pauses

Silent pauses between speech segments were divided into two major categories: within-turn (WT) pauses and between-turn (BT) pauses. WT pauses were silent pauses that occurred within a participant’s turn (i.e., in the absence of a speaker change). BT silent pauses occurred when a speaker changed from a confederate to a participant without overlapping. This measure was used to estimate participants’ latency to respond, as previous studies showed that autistic individuals take longer to respond than NT comparison individuals [71]. BT pauses from participant to confederate (i.e., the confederate’s latency to respond) were not analyzed in this study, because they did not differ by group. Silences before or after the conversations began or ended were excluded from analysis. Duration of each speech segment and silent pause was measured, and counts of speech segments and silent pauses per minute were also calculated. We also measured total and mean duration of speech segments and WT and BT silent pauses per speaker.

In addition to duration features, we measured the total number of words produced by participants. We calculated participant speech rate (number of words per minute) by dividing the total number of words by the sum duration of speech segments plus WT and BT pause duration and multiplying this value by 60. Lastly, we pitch-tracked the audio files using the Robust Algorithm for Pitch Tracking [72] with a pitch range of 75–400 Hz and extracted pitch values per 5 ms. Raw pitch values from voiced frames in participants’ speech segments were converted to semitones (st) using the 10th percentile of each individual’s pitch range as a baseline (st = log_2_(f0/baseline) * 12) in order to control for physiological differences between boys and girls and between younger and older children. These converted pitch values were used to calculate pitch range in median absolute deviation from median (MAD).

In total, 15 speech features were analyzed in this study: participants’ total speech duration, mean speech segment duration, number of speech segments per minute, total overlap duration, total interrupting duration, total being-interrupted duration, total BT pause duration, mean BT pause duration, number of BT pauses per minute, total WT pause duration, mean WT pause duration, number of WT pauses per minute, total number of words, speech rate, and pitch range. The durational measures analyzed in this study comprise a near-comprehensive list of temporal measures that can be examined in natural conversations, while the other three measures (total number of words, speech rate, and pitch range) were included to enhance comparability with prior literature focused on autistic speech.

### Statistical analysis

We first implemented Shapiro–Wilk normality tests and Levene’s tests to check whether the variance in participants’ speech features met the requirements necessary for parametric statistical approaches. When a given speech feature met the requirements necessary for parametric testing, we built an analysis of covariance (ANCOVA) model, where our language variable was the dependent variable and group (autistic girl, autistic boy, NT girl, NT boy) was the independent variable. ANCOVA models were employed in this study because our goal was to compare all groups to each other. Participants’ chronological age was covaried in the model, because the groups significantly differed on age. When a given speech feature failed to meet the requirement for parametric testing, we used the log-transformed values of those speech variables to build ANCOVA models. The directionality of significant group differences identified by our ANCOVA models was assessed via Tukey-corrected pairwise post hoc comparisons on covariate-adjusted estimated marginal means, using the emmeans package [73] in R [74]. *p*-values were adjusted using a false discovery rate for multiple group comparisons (*n* = 4). We also report the effect size of each comparison using Cohen’s *d*, which was calculated after considering the correlation of the covariate and the output measure using the compute.se package [75] in R. We conducted additional analyses to examine whether any significant difference between autistic boys and girls changed when covarying for SRS-2 scores (durational measure ~ age + SRS-2 total score + sex) using linear regressions, as autistic boys and girls only marginally differed in SRS-2 scores (*p* = 0.051, Table 1). However, these additional analyses did not change the results, so we only reported the results of the main analyses.

### Ethics

This study was conducted at the Center for Autism Research with approval from and oversight by the Institutional Review Board of the Children’s Hospital of Philadelphia. Parents provided written informed consent for their children to participate and for audio/video recordings to be used for research purposes, and children provided verbal assent before collection of language samples.

## Results

### Speech segments

**Fig. 1:**
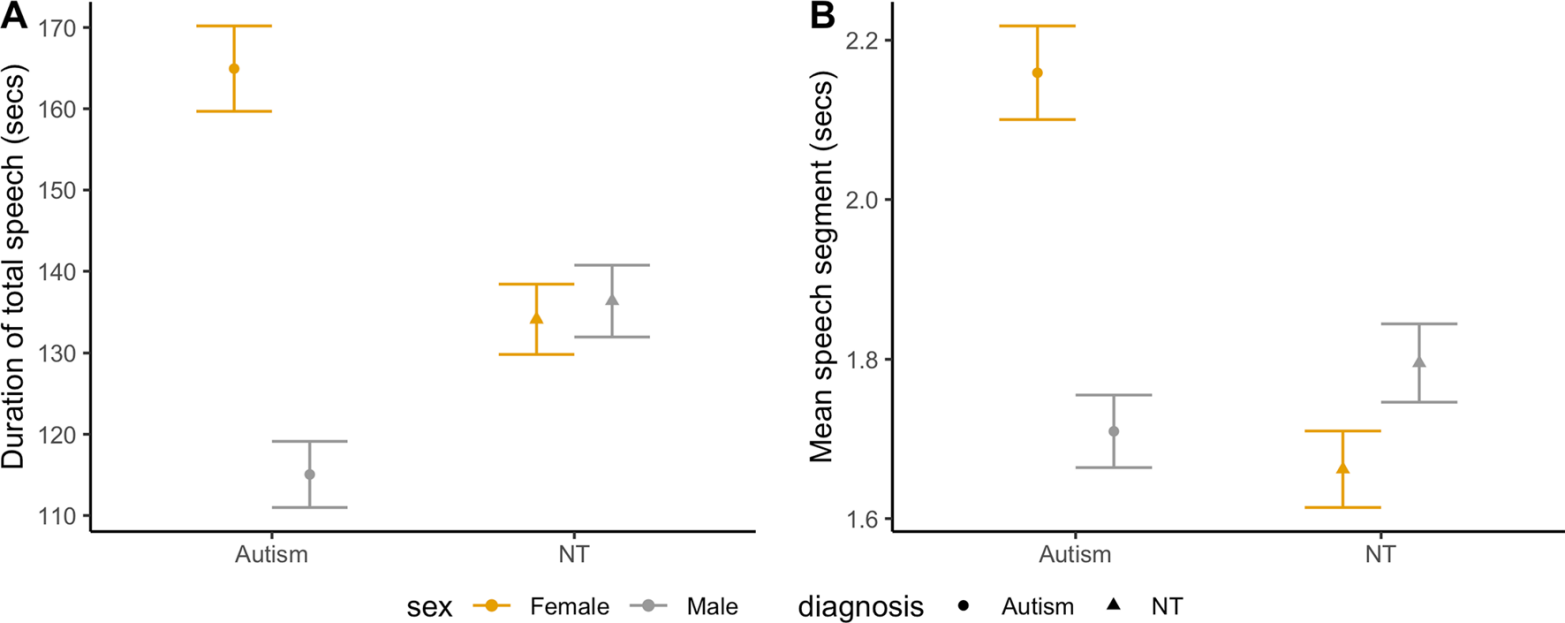
Estimated marginal means and standard errors for total speech duration and mean speech segment duration by group

Participants’ total speech duration differed by group (*F*(3,87) = 4.55, *p* = 0.005, |*d*|= 0.44; Fig. 1A). Autistic girls spoke significantly longer (164.94 $\pm$ 10.51 s) compared to autistic boys (115.06 $\pm$ 8.14 s; *p* = 0.002). Other groups did not differ from one another (NT girls: 134.12 $\pm$ 8.59 s, NT boys: 136.36 $\pm$ 8.78 s; autistic boys vs. NT boys: *p* = 0.127, autistic boys vs. NT girls: *p* = 0.141, autistic girls vs. NT boys: *p* = 0.077, autistic girls vs. NT girls: *p* = 0.074, NT boys vs. NT girls: *p* = 0.86). Mean speech segment duration also differed significantly by group (*F*(3,87) = 4.14, *p* = 0.009, |*d*|= 0.42; Fig. 1B). Autistic girls’ speech segment duration (2.16 $\pm$ 0.12 s) was significantly longer than all the other groups (vs. autistic boys (1.71 $\pm$ 0.09 s): *p* = 0.011; vs. NT boys (1.8 $\pm$ 0.1 s): *p* = 0.038; vs. NT girls (1.66 $\pm$ 0.1 s): *p* = 0.008). Other pairwise comparisons were not significant (autistic boys vs. NT boys: *p* = 0.64, autistic boys vs. NT girls: *p* = 0.73, NT boys vs. NT girls: *p* = 0.5). Counts of speech segments per minute did not differ significantly by group (*F*(3,87) = 2.43, *p* = 0.07, |*d*|= 0.32).

### Overlapping speech

**Fig. 2:**
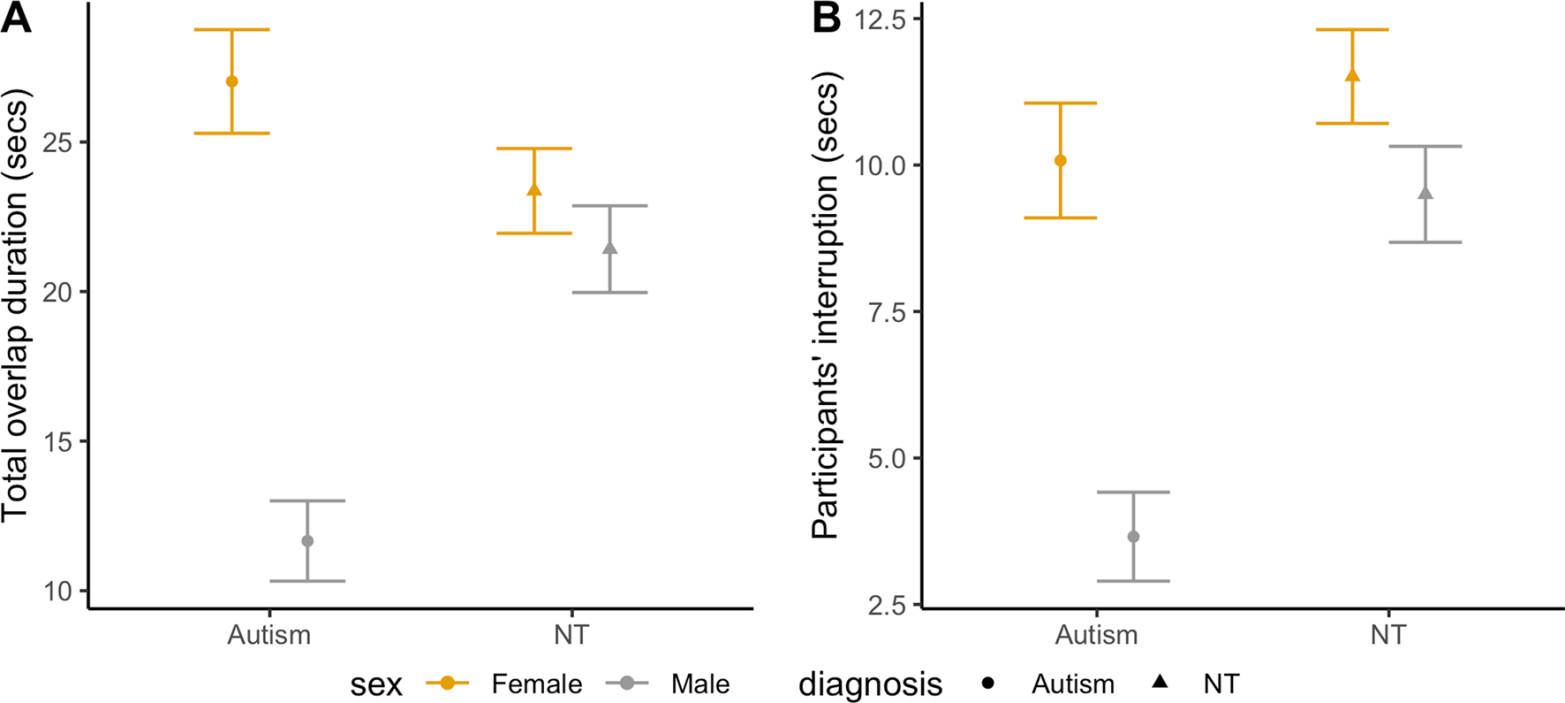
Estimated marginal means and standard errors for total overlap duration and total duration of participants’ interruptions by group

Total duration of overlapping speech also differed by group (*F*(3,87) = 4.83, *p* = 0.004, |*d*|= 0.41; Fig. 2A). Conversations between confederates and autistic boys had a significantly shorter overlapping speech duration (11.66 $\pm$ 2.69 s) than other groups (vs. autistic girls (27.02 $\pm$ 3.47 s): *p* = 0.005; vs. NT boys (21.42 $\pm$ 2.9 s): *p* = 0.035; vs. NT girls (23.36 $\pm$ 2.83 s): *p* = 0.013). Also, the duration of overlapping speech instances that were initiated by participants differed significantly by group (*F*(3,87) = 4.62, *p* = 0.005, |*d*|= 0.4; Fig. 2B). Autistic boys interrupted confederates’ speech less (3.66 $\pm$ 1.52 s) than all other groups (vs. autistic girls (10.08 $\pm$ 1.96 s): *p* = 0.025; vs. NT boys (9.5 $\pm$ 1.64 s): *p* = 0.025; vs. NT girls (11.51 $\pm$ 1.6 s): *p* = 0.004). All the other group comparisons were not significant, and the total duration of overlapping speech initiated by confederates did not differ by group (*F*(3,87) = 2.36, *p* = 0.077, |*d*|= 0.31).

### Between-turn (BT) and within-turn (WT) pauses

**Fig. 3:**
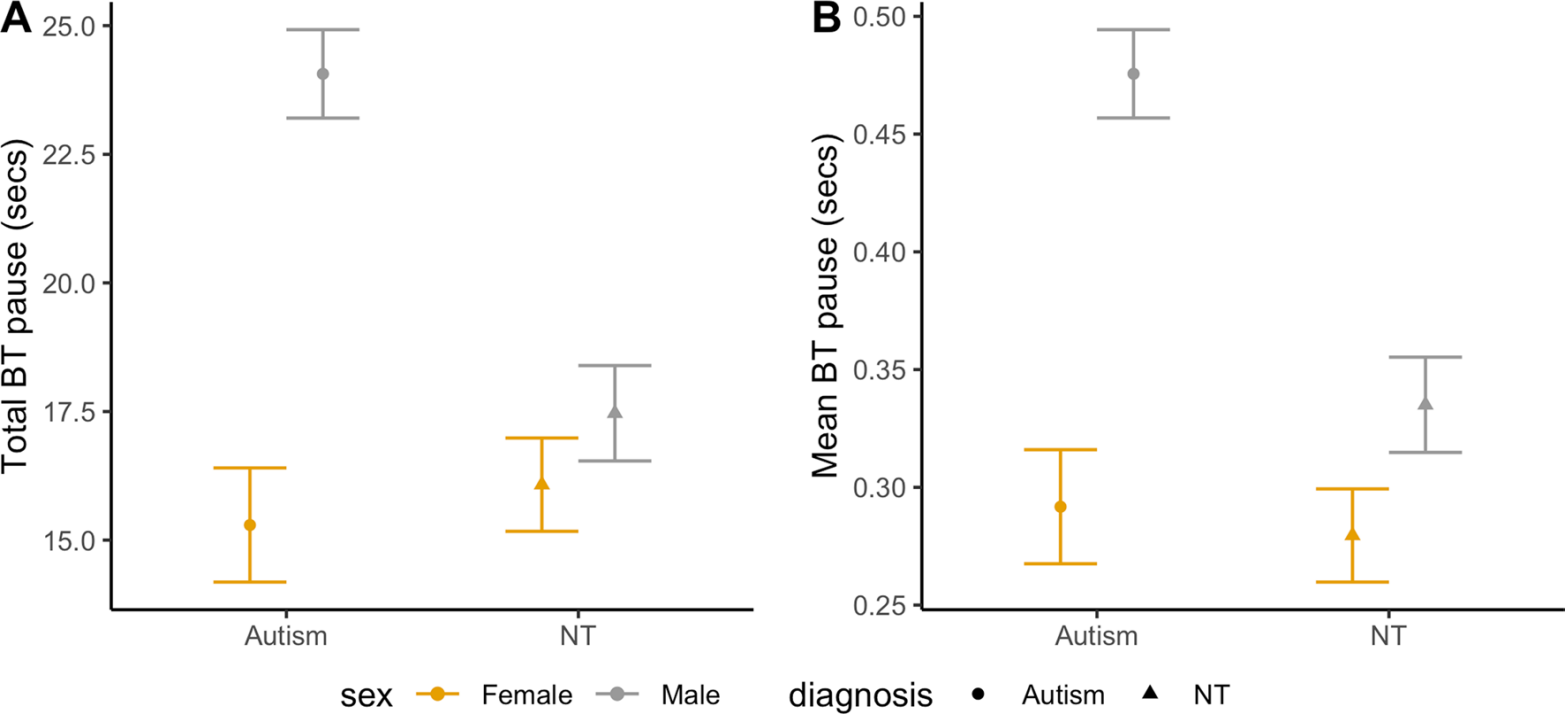
Estimated marginal means and standard errors of duration measures of participants’ total and mean BT silence pause segment durations by group

The total duration of the participants’ latency to respond, which was measured using the duration of BT silent pauses from confederate to participant, differed significantly by group (*F*(3,87) = 4.57, *p* = 0.005, |*d*|= 0.43; Fig. 3A). In particular, autistic boys’ total latency duration (24.06 $\pm$ 1.72 secs) was significantly longer than those of the other groups (vs. autistic girls (15.29 $\pm$ 2.22 secs): *p* = 0.009; vs. NT boys (17.47 $\pm$ 1.85 secs): *p* = 0.025; vs. NT girls (16.08 $\pm$ 1.81 secs): *p* = 0.009). Other groups did not differ significantly from one another. The mean duration of confederate-to-participant BT pauses also significantly differed by group (*F*(3,87) = 4.99, *p* = 0.003, |*d*|= 0.45; Fig. 3B). Autistic boys produced longer BT pauses (0.48 $\pm$ 0.04 s) on average compared to other groups (vs. autistic girls (0.29 $\pm$ 0.05 s): *p* = 0.012; vs. NT boys (0.34 $\pm$ 0.04 s): *p* = 0.029; vs. NT girls (0.28 $\pm$ 0.04 s): 0.004). The rate of BT pauses per minute did not differ by group (*F*(3,87) = 1.44, *p* = 0.236, |*d*|= 0.25). Groups did not differ in WT pause measures, including the total and mean WT pause durations (total: *F*(3,87) = 1.16, *p* = 0.328, |*d*|= 0.22; mean: *F*(3,87) = 1.99, *p* = 0.122, |*d*|= 0.29) and the number of WT pauses per minute (*F*(3,87) = 2.45, *p* = 0.069, |*d*|= 0.33).

### Total number of words, speaking rate, and pitch range

**Fig. 4:**
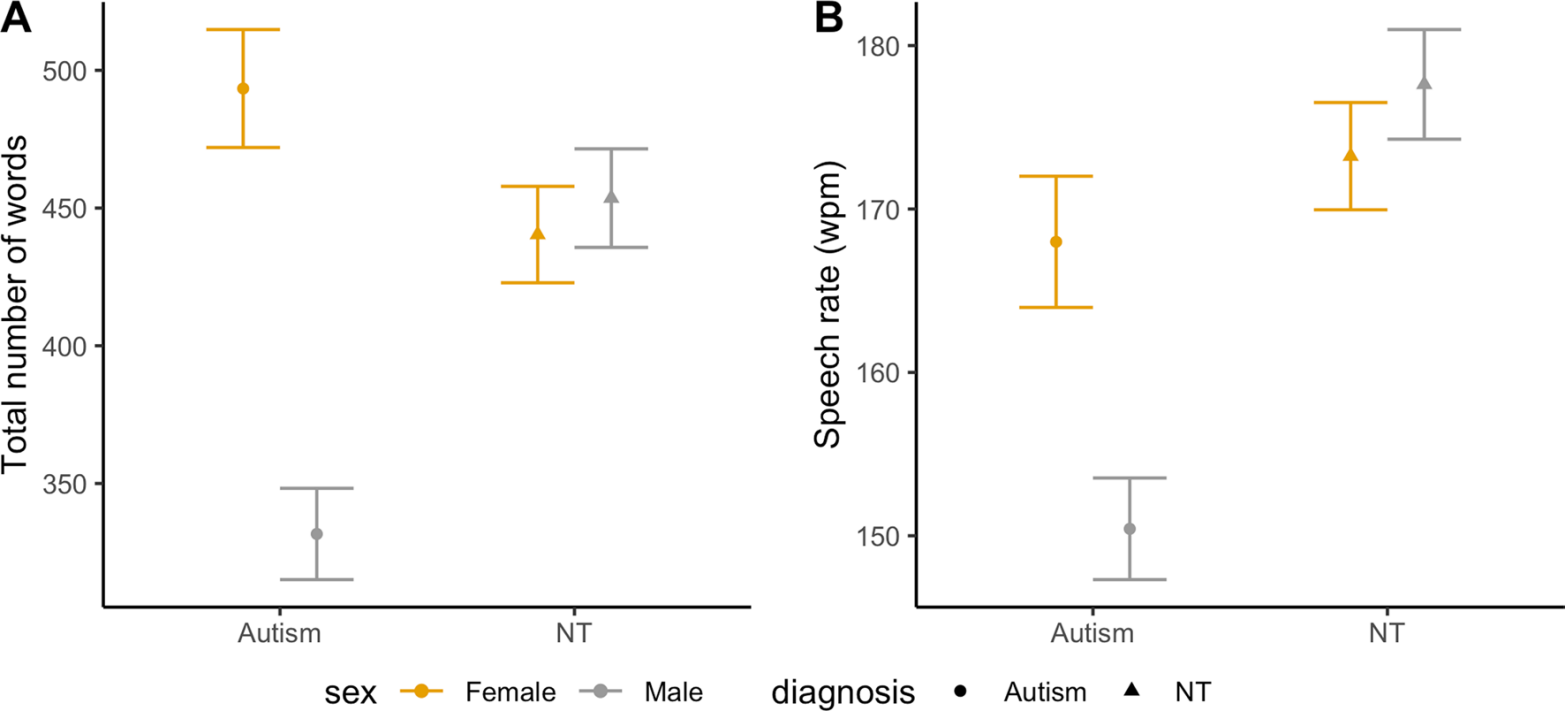
Estimated marginal means and standard errors for total number of words and speech rates by group

The total number of words produced by participants differed significantly by group (*F*(3,87) = 3.52, *p* = 0.018, |*d*|= 0.37; Fig. 4A). Autistic boys produced fewer words (331.69 $\pm$ 33.18) than autistic girls (493.4 $\pm$ 42.83, *p* = 0.026) and NT boys (453.6 $\pm$ 35.79, *p* = 0.049), but not NT girls (440.37 $\pm$ 35). Groups significantly differed on speech rate (*F*(3,87) = 3.3, *p* = 0.024, |*d*|= 0.33; Fig. 4B). Autistic boys spoke more slowly (150.43 $\pm$ 6.22 words per min) than NT girls (173.23 $\pm$ 6.56 words per min, *p* = 0.046) and NT boys (177.63 $\pm$ 6.71 words per min, *p* = 0.027). Autistic girls’ speech rate (167.99 $\pm$ 8.03 words per min) did not differ from any other groups. Groups did not differ on pitch range (*F*(3,87) = 1.21, *p* = 0.311, |*d*|= 0.23). We also investigated other common pitch values, such as means, medians, and standard deviations, as well as interquartile ranges (75th percentile—25th percentile). There were no significant group differences in these values.

## Discussion

For autistic children and adolescents, conversing with a novel adult in an unfamiliar setting without a specific topic to discuss is a challenge that mirrors the frequent unstructured interactions of day-to-day life. In this study, we observed subtle differences in conversational tempo, talkativeness, and responsiveness that distinguished the speaking patterns of autistic girls and boys. Driven by slower responding in autistic boys and more and faster speaking by autistic girls, these objective differences could partly explain why autism is routinely overlooked in girls and women, whereas it is more frequently noticed in boys and men. For example, parents, teachers, and physicians may perceive autistic girls’ speech as being typical (and not classically “autistic-sounding”), resulting in reduced referral rates and subsequent underdiagnosis compared to autistic boys who more closely fit the prototypical male-referenced autism mold. The current study highlights that it is not only critical to understand how the temporal dynamics of conversation play out in autism writ large, but also to explore sources of behavioral *heterogeneity* in verbal communication—such as sex—that could prove useful for enhancing autism detection and informing the development of personalized clinical supports.

### Talkativeness and tempo

At the outset, we hypothesized that autistic girls would speak more with a novel interlocutor than autistic boys, and our results confirmed this initial hypothesis. However, we also found that autistic girls produced longer speech segments than NT peers (both girls and boys), adding new richness and nuance to our understanding of how autism manifests in girls. Pending further research that incorporates factors like anxiety and temperament, longer speech segments could be a potential marker of autism in verbally fluent girls without intellectual disability.

Why might autistic girls produce the longest speech segments, on average, of all the groups in our sample? There are at least three plausible explanations: First, it is possible that by speaking more and actively participating in the conversation, autistic girls in our sample may, consciously or not, be attempting to “mask” or “camouflage” their difficulties with social communication. This explanation is consistent with prior research demonstrating that autistic girls are more likely to present with increased levels of talkativeness in naturalistic conversations compared to autistic boys [24]. However, the fact that autistic girls produced longer speech segments than NT peers could also reflect inaccurate camouflage that “overshoots the mark.” This interpretation aligns with previous observations that girls and women with autism report engaging in more effortful social masking than boys and men with autism [23], and evidence of potentially incomplete or inaccurate speech camouflaging behaviors in other studies [30]. Second, girls with autism may have genuinely stronger social motivation than boys with autism [34], leading to greater social interest and investment in the interaction that manifests as more talking compared to other groups. Notably, responses that are too short and blunt during natural conversations could be interpreted as the speaker being uninterested or, depending on the context, be perceived as socially inappropriate, so it is possible that autistic girls tried to avoid this awkward situation and demonstrate social interest by talking longer to novel interlocutors. Thus, while producing more and longer speech segments could be a useful way for autistic girls to facilitate smooth social interactions and meet gendered societal expectations about female behavior, these same skills in the context of a historically male-referenced clinical prototype for “autism” could ultimately hinder early detection and delay access to much-needed clinical supports. Finally, because this study did not investigate conversation topics, it is possible that autistic girls talked primarily about their own special interests—to an even greater extent than autistic boys—and were therefore motivated to produce the longest speech segments. Future research is necessary to determine whether the observed differences in autistic girls’ total speech time and mean speech segment duration during conversations was due to active “camouflaging,” strong social motivation, special interests, or a combination of these factors.

Compared to autistic girls, autistic boys produced shorter speech segments and spoke for less time overall. Communication can be successful, according to classic Gricean pragmatics [76], when speakers cooperate with one another by following four maxims: the maxims of quantity (be informative), quality (be truthful), relation (be relevant), and manner (be clear). The maxim of quantity, in particular, states that speakers should be as informative as possible when having conversations and give as much information as required for successful communication, but not more than necessary. From this perspective, it could be argued that both boys and girls with autism violated the maxim of quantity—however, *how* they violated this maxim differed by sex. Boys with autism might have offered less information by talking less than NT children, whereas girls with autism might have provided more information than necessary by talking longer. This violation of a basic conversational maxim could result in both boys and girls with autism sounding “atypical” to expert clinicians, which might explain the insignificant sex difference in autistic girls’ and boys’ clinician-rated social communication skills. We did not analyze the conversations qualitatively to assess children’s adherence to Grice’s conversational maxims, so this question is ripe for investigation using qualitative discourse analyses in future research. Previous studies also showed that children with autism produced fewer words in total [41] and spoke more slowly than NT children [48]. However, our findings suggest that *only boys* with autism were more likely to produce fewer words in total and speak more slowly than NT children, whereas autistic girls did not differ from NT girls or boys in the total number of words produced, nor in speaking rate.

### Interruptions

Overlapping speech frequently occurs in natural conversations. Prior research on NT adults shows that 40–54.1% of between-turn intervals included overlapping speech with an average duration of 280–610 ms [77–79]. Given that overlapping speech is natural and common, the fact that autistic boys had less overlapping speech overall than the other groups could indicate that the conversational flow between autistic boys and their interlocutors was “unnatural” per NT conversational norms. This might be one reason why autistic boys were found to make less-good first impressions than autistic girls after a brief conversation [10]. Total duration of overlapping speech produced by autistic girls, in contrast, did not differ from NT children. Thus, normative overlapping speech patterns could represent another subtle form of masking employed by autistic girls as they attempt to socially blend in with their peer groups, and which, when combined with other subtle factors, could contribute to reduced referral rates and underdiagnosis.

Prior research suggests that there are at least two types of overlapping speech: one for interrupting others’ turns and the other for expressing affirmation or sympathy by collaboratively completing others’ utterances (i.e., backchanneling) [80]. In this study, we did not qualitatively analyze which type of overlapping speech children with autism produced, so it remains unclear whether the ratio of interrupting to cooperative overlapping speech differs between NT children and autistic children and how overlapping speech produced by autistic children may be perceived by naïve listeners. This question requires future research.

### Pauses

It is common for conversations with novel interlocutors to contain some lengthy silent pauses, because the speakers do not know each other well. However, frequent long pauses during conversations may be perceived as awkward or indicate poor social communication skills [81, 82]. Interestingly, many previous studies showed that autistic children responded more slowly and produce more frequent pauses than NT peers when replying to interlocutors during naturalistic conversations [48, 51, 83–85]. However, because most prior studies included predominantly male samples, we hypothesized that autistic boys might have driven these effects and thus would demonstrate longer latency to respond than autistic girls in the current study. Our results confirmed this hypothesis, showing that boys with autism produced longer BT pauses on average than girls with autism, and their total BT pause duration was longest among all groups. Autistic girls, in contrast, did not show this pattern and did not differ from NT children in terms of BT pause duration and frequency. This pattern of results suggests that a widely accepted finding in the literature (i.e., that autistic children take longer to respond than NT children) may have been an artifact of unbalanced research samples that did not include sufficient numbers of autistic girls, and thus may only be expected to hold true only for autistic boys. Finally, the fact that girls’ BT pause patterns did not differ by diagnosis suggests that engaging in normative temporal dynamics during naturalistic conversations may be a particular area of strength for verbally fluent autistic girls.

Taken together, the findings reported here illustrate that even simple language measures like total number of words, pauses, speech duration, and speaking rate can help define sex-related social phenotypes in autism, and even when autistic girls and boys are matched on autism symptoms as rated by expert clinicians, they may differ from one another and from NT peers in unique and informative ways. In fact, the specific verbal communication strategies demonstrated by autistic girls in this study, such as shorter and fewer BT pauses, could be also employed by other people with verbal communication difficulties who desire to improve their conversational outcomes.

## Limitations and future directions

Despite numerous strengths, the current study also has limitations that should be considered when interpreting our findings. First, the present sample included verbally fluent children and adolescents aged 6–15 years without intellectual disability, and thus our results may not generalize to samples of younger children, adults, individuals who are not verbally fluent, or individuals with verbal IQ estimates below 70. Second, despite being one of the larger studies of natural conversational behavior in autism, the sample we report here is nonetheless limited, with effect sizes for each variable ranging from small to medium. Therefore, these results should be interpreted with caution, and the current findings should be replicated in future research studies with larger and more diverse samples. Notably, due to the high rates of missed or misdiagnoses in autistic girls [13], it is unclear whether the pattern of results we report will apply to the population of girls who are autistic but have not yet been detected using standard diagnostic methods and referral practices. Boys and girls in this sample were predominantly White and non-Hispanic, limiting our ability to assess how social language might differ in non-White and/or Hispanic children, and highlighting the need for future research in cohorts with greater racial and ethnic diversity. The socioeconomic diversity of our sample as measured by mothers’ education level was also limited. Given that conversational abilities are directly influenced by socioeconomic factors [86–88], future research on social language in autism should include measures that characterize socioeconomic status and investigate linguistic variability across this dimension.

Our methods and approach had several limitations as well. First, our sample was cross-sectional, so it is unclear whether these results will persist over developmental time. We are currently addressing this shortcoming in a new study, wherein we collect longitudinal speech samples from autistic children and NT children over the course of seven research visits. Another limitation is that all study conversations were conducted between children and a novel young adult confederate. This study design allowed us to examine how children might behave in a communication setting wherein they encounter a novel adult; however, future research is needed to examine how children communicate with people with whom they are more familiar, including family and same-aged peers, and how this differs from their behavior during conversations with adult strangers. In a new study to address this limitation, we are thus exploring the effect of conversing with different interlocutors. To our knowledge, no previous study has examined whether certain conversational features (e.g., longer latency to respond), might be perceived as more atypical than other features (e.g., longer-than-average speech segments). This could be an interesting question to explore in the context of a perception experiment. Lastly, it is important to note that many of our young adult interlocutors were female. Since the sex of a conversation partner likely influences child behavior, future research should aim to control more closely for interlocutor sex and consider implementing confederate–participant matching as well as mismatching on this variable.

## Conclusions

The results of this study contribute to a growing literature aimed at sharpening our conceptualization of autism in girls by quantifying subtle differences in conversational language. By investigating simple durational measures that underlie the temporal dynamics of conversation, such as total speech time and latency to respond, we showed that autistic girls and boys have distinct language profiles comprised of many small differences. Importantly, some previously reported differences in language that were thought to characterize *all* autistic children, such as slower conversational response times during conversations, might instead apply primarily to autistic boys. A major implication is that autistic girls may be more likely missed or misdiagnosed due to poorly understood social interaction profiles—including spoken language—that vary by sex and are just beginning to be systematically explored. Understanding sex-differentiated conversational language profiles could shed light on the clinical heterogeneity that complicates early identification and diagnosis in autism, and could inform the development of supports to meet the unique needs of girls and women. Finally, we propose that all future clinical trials, intervention studies, and basic research efforts be required to maintain samples that are sufficiently powered to detect potential sex differences in autism, so that autistic girls and women can begin to benefit equitably from the scientific process.

## Supplementary Information

**Additional file 1**: Sample excerpts from participants’ conversation. 

## Data Availability

The datasets generated and/or analyzed during the current study are not publicly available due to embedded protected health information in conversations with minors, but are available on reasonable request with proper safeguards.
