# Supplementary material for: Sex differences in the temporal dynamics of autistic children’s natural conversations
Source: Mol Autism. 2023 Apr 6;14:13. doi: 10.1186/s13229-023-00545-6 (PMC10080787; doi:10.1186/s13229-023-00545-6)
Supplement: Supplementary file 1 — Additional file 1: Sample excerpts from participants’ conversation. [file 13229_2023_545_MOESM1_ESM.docx]

Appendix – Sample excerpts from participants’ conversation

1. A boy with autism (age: 13 years)

Confederate: Are you doing anything fun this summer?

Participant: I am going to a camp.

Confederate: Oh cool. What camp is it?

Participant: The YMCA.

Confederate: Okay.

Confederate: What do you like to do at camp?

Participant: Swimming.

Confederate: Swimming? Me too. I love swimming.

Confederate: Um. What’s your favorite – Do you have a favorite swimming stroke?

Participant: Um. No.

Confederate: No {laugh}? Just general swimming? Do you have friends at camp?

Participant: Mm-mm.

1. A girl with autism (age: 12 years)

Confederate: Do you like plays for school or?

Participant: Yeah there – The school play was recently. I didn’t really like it, but I went along with it. {laugh}

Confederate: Yeah which, which one was it because I did some of that?

Participant: Schoolhouse Rock.

Confederate: Oh, okay.

Participant: {laugh} It was just like – because like we – It was so corny and cheesy so just had to be like {laugh}.

Confederate: Yeah.

Participant: And that was okay um {laugh}. The school play last year I liked better.

Confederate: Yeah.

Participant: Last year we did Lion King and that was really fun.
